# How Does Air Pollution Influence Housing Prices in the Bay Area?

**DOI:** 10.3390/ijerph182212195

**Published:** 2021-11-20

**Authors:** Minmeng Tang, Deb Niemeier

**Affiliations:** 1 Department of Land, Air and Water Resources, University of California, Davis, One Shields Ave, Davis, CA 95616, USA; 2 Department of Civil and Environmental Engineering, University of Maryland, 1173 Glenn Martin Hall, College Park, MD 20742, USA; niemeier@umd.edu

**Keywords:** air pollution, housing price, instrumental variable, spatial autocorrelation, spatial lag model

## Abstract

In this paper we examine the effects of localized air pollution measurements on the housing prices in Oakland, CA. With high-resolution air pollution measurements for NO, NO_2_, and BC, we can assess the ambient air quality on a parcel-by-parcel basis within the study domain. We combine a spatial lag model with an instrumental variable method to consider both the spatial autocorrelation and endogeneity effects between housing prices and air pollution concentrations. To the best of our knowledge, this is the first work in this field that combines both spatial autocorrelation and endogeneity effects in one model with accurate air pollution concentration measurements for each individual parcel. We found a positive spatial autocorrelation with housing prices using Moral’s I (value of 0.276) with the total sample number of 26,386. Somewhat surprisingly, we found a positive relationship between air pollution and housing prices. There are several possible explanations for this finding. Homeowners in high demand, low-stock housing areas, such as our study, may be insensitive to air pollution when the overall ambient air quality is relatively good. It is also possible that under clean air conditions, low variability in pollutant concentrations has little effect on property values. These hypotheses could be verified with more high-resolution air pollution measurements with a diversity of regions.

## 1. Introduction

Air pollution is not only a major global risk resulting in high incidences of illness and deaths [1,2], but can also produce external damages to different economic sectors, including manufacturing, agriculture, transportation, and utilities [3]. In the U.S., air pollution costs were roughly equivalent to about 5% of the yearly gross domestic product (GDP) in 2014 [4]. One sector we might expect to be highly sensitive to air quality is housing, and there are a number of studies both nationally and globally focusing on the relationship between air quality and housing prices.

The literature mainly relies on the construction of the hedonic price models to evaluate the effect of air pollution on housing prices. The hedonic price model, commonly used in economy, focuses on the relation between price and other corresponding features [5]. It has been widely used in the housing market studies [6,7]. Some new regression methods, such as neural network, quantile regression, and semi-log regression, have also been applied in housing price prediction studies [8]. For the studies focusing on how air pollution influences housing prices, we can divide the body of research based on the approach. The first category uses an instrumental variable to address endogeneity effects and frequently uses a variable that is not related to housing prices but directly related to air pollution as the instrumental variable to determine the exogeneous part of the variability from air pollution [9,10]. The endogeneity effect means correlation between the explanatory variable and the error term, which leads to the biased estimates using the ordinary least square estimation method. The second group uses spatial econometric models and hedonic price models to understand air pollution’s influence on housing prices, accounting for spatial autocorrelation of housing prices. Spatial autocorrelation is a term that is used to describe the systematic spatial variation in a variable. For example, positive spatial autocorrelation, which is a more common situation, means that sites that are located close together tend to have similar values. The most common spatial hedonic models are the spatial lag model (SLM) [11,12], spatial error model (SEM) [11,12], spatial Durbin model (SDM) [13], geographically weighted regression (GWR) [14,15], and quantile regression models (QRM) [15]. The results from the literature are inconclusive: some of the studies conclude that air pollution concentrations do not significantly influence housing prices [11,12,16], while others find that air pollution concentrations negatively and significantly influence housing prices [10,17,18,19,20].

Previous studies have produced inconclusive findings, in part because there were limitations to the approaches. For example, nearly all the studies consider only spatial autocorrelation or endogeneity effects. Most studies rely on Moran’s I to measure spatial autocorrelation [21], and results from cities in both China and the U.S. suggest that there are positive and significant spatial autocorrelations in housing prices [22,23]. When air pollution is added to the mix, the endogeneity effect on housing prices results in model estimation and causal inference biases [9,15,17]. We depart from previous studies by constructing a hedonic price model combining both spatial autocorrelation and endogeneity effects to examine the relationship between housing prices and air pollution. To the best of our knowledge, this is the first study combining these two effects to comprehensively understand how air pollution influences housing prices. We also introduce high-resolution air pollution mapping data into housing valuation studies. Prior research relied on air pollutant data from a limited number of stationary monitors to underpin estimation for a large region or a city. Our high-resolution mobile-based air pollution mapping data cover every street within the study domain, which allows us to draw on much more accurate ambient air quality measurements for each property.

## 2. Materials and Methods

### 2.1. Study Area

**Figure 1:**
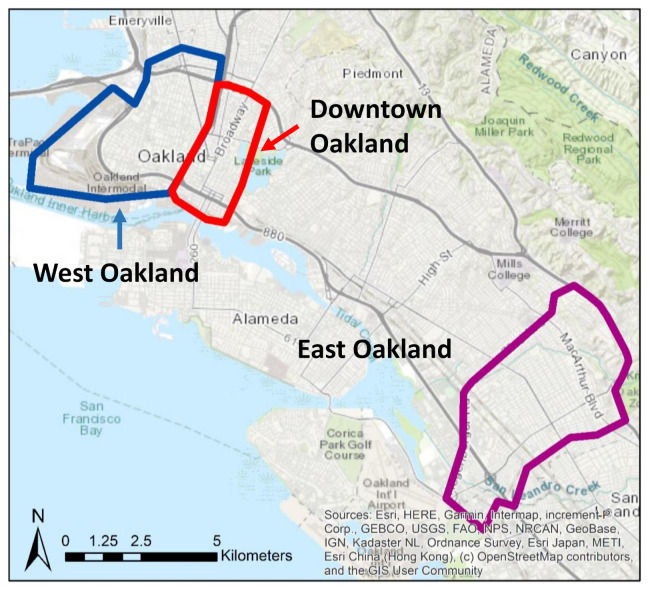
Study domain.

Our study domain includes three major areas within Oakland, California: West Oakland (WO), Downtown Oakland (DO), and East Oakland (EO) (Figure 1). The WO and DO areas together cover about 15 km^2^, with residential, commercial, and industrial blocks, and the EO area covers about 15 km^2^ with a mix of industrial and residential blocks. The WO and DO areas have a total population of about 25,000, and the EO area has a total population of about 58,000 [24].

### 2.2. Pollutant Concentration and Housing Valuation Data

Two Google street view mapping vehicles, carrying Aclima environmental intelligence sensors, were deployed in the study area between June 2015 and May 2016. The dataset covers the measurements of weekday daytime concentrations of black carbon (BC), nitric oxide (NO), and nitrogen dioxide (NO_2_) with one second temporal resolution within the study area (Figure 1). A mobile-based data reduction and aggregation algorithm was developed by Apte et al. [25] to average the instantaneous measurements into median annual weekday concentrations with 30 m resolution [26]. We used the high-resolution air pollution concentration product from Apte et al.’s [25] supporting information as ambient air pollution measurement in our study. Since meta-analyses have demonstrated that the spatial extent of mobile sources is in the order of 100–400 m for particulate matter and 200–500 m for NO_2_ [27,28], we selected 400 m as the buffer size and calculated the mean air pollution concentrations within the buffer area of each property to represent the ambient air pollution concentrations. We also calculated air pollution concentrations with a 100 m buffer and without any buffer. The results and conclusions were the same as those produced with the 400 m buffer. For the purposes of this paper, we used the 400 m buffer air pollution concentrations calculated to ensure that we incorporate proximate roadway-generated air pollution.

**Figure 2:**
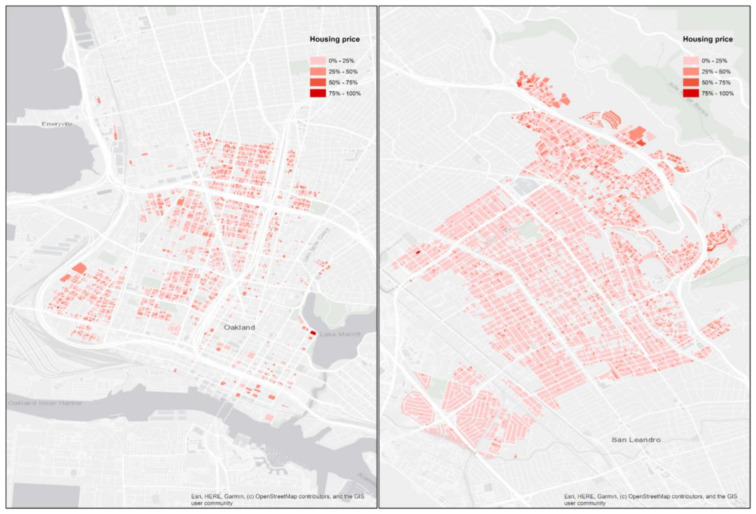
Housing price spatial distribution in the study domain.

The housing valuation data (shown in Figure 2) were provided by Estated, Inc. (Boulder, CO, USA), (https://estated.com/ accessed on 13 August 2020), and include land, improvement, and total value for every property within our study domain. The value of each property is calculated based on tax assessment as provided by the county assessor. For each property, the detailed structure information includes year built, stories, room counts, parking type, construction type, and total area. Finally, sociodemographic variables at the census tract level influencing housing price, including population density, income, and non-employment rate, were assembled using the 2016 American Community Survey.

### 2.3. Methods

Following Kim et al.’s study [16], in which the SLM model specification outperformed SEM on housing data in Korea, we constructed a spatial lag model (SLM) with an additional instrumental variable to include both the spatial autocorrelation and endogeneity effects (Equation (1)):

(1) $$y=X\beta +\lambda Wy+\varepsilon ,\;\varepsilon \sim N\left(0,\sigma^{2}\right)$$

where *y* is the logarithm of housing price, *X*’s are independent variables including an instrumental variable, *β* are the estimated coefficients, *W* is the non-stochastic spatial weight matrix, *Wy* represents the spatial lag of the dependent variables, and *ε* is the error term. For the spatial weight matrix, there are no widely accepted spatial structures for housing price data, but some studies use the queen contiguity weighting matrix since it is representative for contiguity-based weighting matrices [12]. Therefore, we used the queen contiguity weighting matrix.

To address the endogeneity concern between housing prices and air pollution concentrations, we combined the instrumental variable (IV) method together with the SLM. We used the mean of the median vehicle speed within the buffer area as the instrumental variable, which is positively related to air pollution concentrations but is not correlated with housing prices.

The spatial lag term in Equation (1) is an endogenous variable, and the instrumental variable is an additional endogenous variable, which can result in difficulty in estimating the model coefficients due to the extra endogenous variable. We used a two-step generalized moments (GM) and instrumental variable (IV) method to estimate the coefficients in Equation (1) [29,30,31,32,33]. All the calculations were conducted in R [34] and the two-step GM/IV method is available in *sphet* package with function *spreg* [35,36].

## 3. Results and Discussion

### 3.1. Variable Distribution

**Figure 3:**
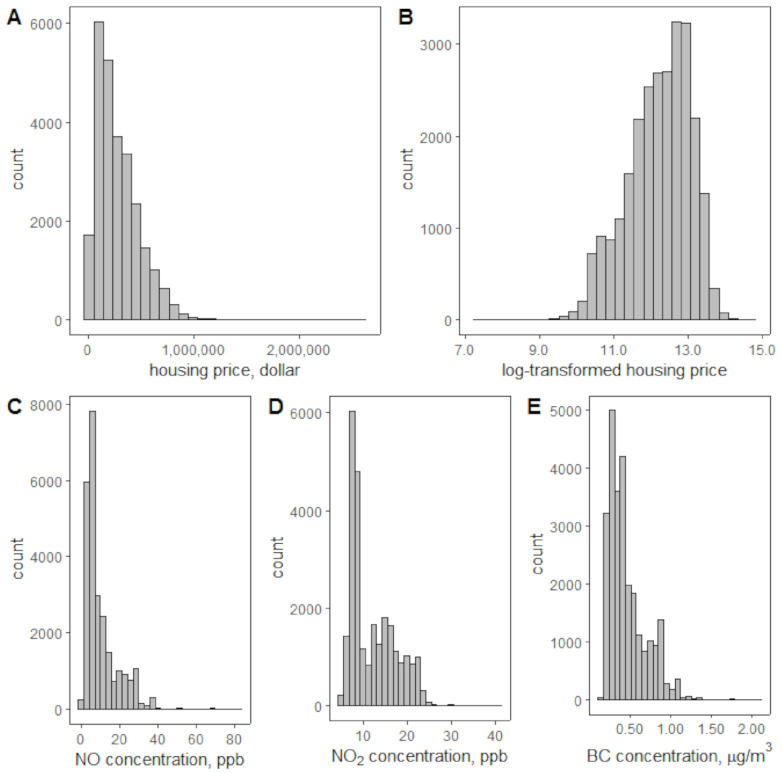
Distributions of housing prices (**A**), logarithm transformed of housing prices (**B**), and concentrations of NO (**C**), NO_2_ (**D**), and BC (**E**).

Housing prices are not normally distributed (Figure 3A), so we applied the logarithm transformation of housing prices (Figure 3B). The NO, NO_2_, and BC concentrations in Figure 3C–E are the average of measurements within the 400 m buffer of each parcel. We also tested using the logarithm transformation of NO, NO_2_, and BC concentrations as input to our model, which obtained very similar results as using the concentrations without transformation. Therefore, no transformation was applied to the NO, NO_2_, and BC concentrations. Most parcels have NO concentrations less than 40 ppb, NO_2_ concentrations less than 25 ppb, and BC concentrations less than 1.5 µg/m^3^. The summary statistics of housing prices and air pollution concentrations are shown in Table 1 below.

### 3.2. Spatial Autocorrelation

**Figure 4:**
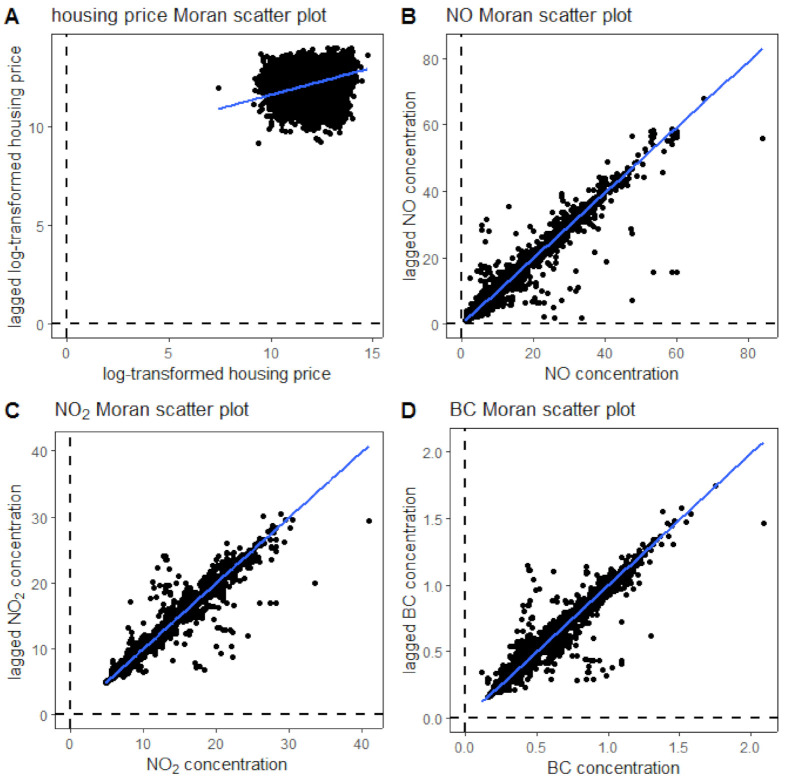
Moran’s I scatter plots of housing prices (**A**), NO (**B**), NO_2_ (**C**), and BC (**D**) concentrations (blue lines are the linear regression lines between variables and the lagged variables; the slopes of blue lines are the Moral’s I statistic).

We used the Moran scatter plot to examine the spatial autocorrelation of housing prices and three air pollutants within the study domain (Figure 4). For the Moran scatter plot, it shows the relation between the spatially lagged variable and the original variable, which suggests how housing prices and air pollutant concentrations are related to their surrounding neighbors. The Moran’s I test is commonly used in geography-related fields to quantify spatial autocorrelation. For the Moran’s I test, the test statistic is represented by the slope of the fitted line in the Moran scatter plot (Figure 4), which measures how one object is similar to its surroundings. We also used the permutation-based random Moran’s I test, which uses the Monte-Carlo simulation method to randomly shuffle the data and calculate the Moran’s I statistic for each random shuffle and compare it with the actual Moran’s I statistic. The results of both Moran’s I tests for housing prices and the three pollutants are shown in Table 2. The housing price has a Moran’s I value equal to 0.276, suggesting a positive spatial autocorrelation. All of the pollutants have Moran’s I values close to 0.99, suggesting highly positive autocorrelation. Therefore, including the spatial autocorrelation term into the hedonic price model is necessary.

### 3.3. Spatial Lag Model Results

The model results of all three pollutants were very similar (Table 3). As expected, the year the home was built negatively influences the housing price, and garage, bath number, total area, and median income positively influence the housing price. Air pollution concentrations positively and significantly influence housing prices, which we will discuss in greater detail in Section 3.4.

### 3.4. Discussion

Based on the coefficients from the SLM model in Table 3, they suggest that all three pollutants (NO, NO_2_, and BC) have a positive and significant effect on housing prices. This is unexpected and we have a few speculations as to why this occurs. First, the air pollution concentrations are low throughout the area. The average concentration of NO is 10.29 ppb, NO_2_ is 12.12 ppb, and BC is 0.46 µg/m^3^. We also included the air pollution concentrations of BC and PM_2.5_ from a stationary monitoring station (Oakland-West site) located in the center of West Oakland (https://ww3.arb.ca.gov/qaweb/iframe_site.php?s_arb_code = 60349, accessed on 15 September 2020). For the stationary data, we calculated the mean values of the hourly measurements between June 2015 and May 2016, which covers the same time range (9 am to 5 pm) of the mobile air pollution measurement in our study. The mean concentrations of BC and PM_2.5_ from the stationary monitor are 0.59 and 8.36 µg/m^3^, respectively. The BC concentrations are close between the stationary monitor measurement and the mobile measurement we used in this study, which provides a general estimate about the PM_2.5_ concentrations across our study domain. Comparing these to the National Ambient Air Quality Standards (NAAQS), the annual standard of NO_2_ is at a level of 53 ppb, and the annual standard of PM_2.5_ is 12.0 µg/m^3^ for a primary source and 15.0 µg/m^3^ for a secondary source [37]. It is possible that when the ambient air quality is relatively clean, affordability dominates the need to pay a housing premium for even cleaner air. Of the 19 papers we found on housing and air quality, 10 papers were relevant to our research. Among these, the findings are mixed (Table 4). Three show insignificant effects of air pollution on housing price. In the remaining seven papers, the air pollution concentrations have significant and negative effect on house prices.

All 10 papers we found used the hedonic price model to study the impact of air pollution on housing prices. Their conclusions were derived from the model coefficients. If the regression coefficient of air pollution is statistically significantly less than zero, air pollution negatively influences housing prices, and if the coefficient is significantly greater than zero, air pollution positively influences housing prices. If the coefficient is not significantly different from zero, air pollution is not significantly influencing housing prices.

As we noted in the introduction, even though all of the papers used the hedonic price model, the authors relied on different methods to emphasize different effects (e.g., instrumental variable (IV), spatial lag model (SLM), spatial error model (SEM), fixed effect, etc.).

In Table 4, among the three studies with insignificant results about air pollution influencing housing prices, they all take the spatial autocorrelation effect into consideration when constructing the hedonic price model. In one study, the authors argue that the insignificant effect of NO_x_ concentrations on housing prices is due to the fact that NO_x_ does not tend to exceed the standard; on the contrary, SO_2_ shows a significant and negative impact on housing prices in the same study, because SO_2_ has exceeded the official air quality standard over a long period of time [16]. The other two studies believe that the insignificant results are caused by either an insufficient degree of efficiency [11] or that the change of air pollution concentration is more important than air pollution concentration itself [12].

In examining the literature, the results are suggestive that air pollution’s effects tend to be insignificant when overall ambient air pollution concentrations are relatively low. In our study, the average air pollution concentrations across all of our sample observations were the lowest among these studies. It is possible that affordability is more important than a housing premium for even cleaner air when the ambient air quality is already good. Therefore, the positive and significant coefficients of air pollution on housing prices may be reasonable in areas with good air quality.

**Figure 5:**
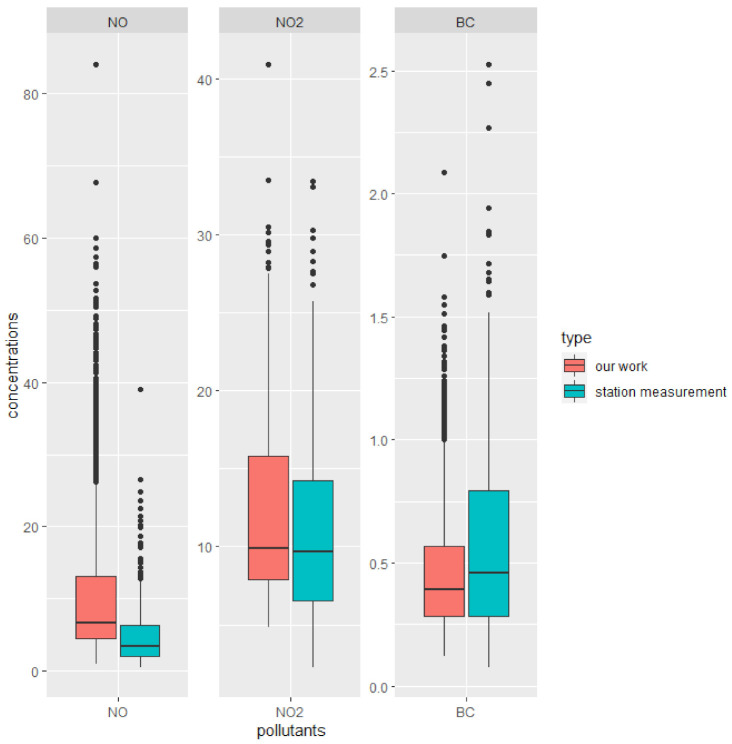
Pollutant distributions’ comparison between our work and one stationary monitor (NO and NO_2_ are in the unit of ppb, BC is in the unit of µg/m^3^).

A second possible reason why we find counterintuitive results may be due to the very low variability in pollutants and housing prices. Within our buffer, standard deviations of NO, NO_2_, and BC concentrations were 8.68 ppb, 5.07 ppb, and 0.23 µg/m^3^, respectively. We compared the distribution of the three pollutants in our study with one stationary monitoring measurement located in the center of West Oakland (WO) in Figure 5. For the stationary data, we used the hourly measurements of NO, NO_2_, and BC from the above-mentioned Oakland-west site, covering the same date and time range of the mobile air pollution measurement in our study. Our data variability is close to the variation of air pollution concentrations at a single location. Low variability may lead to positive and significant coefficients even if the results are not significant.

## 4. Limitations and Conclusions

To the best of our knowledge, this is the first study that combines a spatial lag model with an instrumental variable method to capture both the spatial autocorrelation and endogeneity effects of the relationship between housing prices and air pollution. Our study demonstrated the use of high-resolution air pollution mapping data to quantify the localized ambient air quality at the parcel level, which can largely improve the accuracy of pollution cost valuation. The results of our work have significant policy implications, as the air quality regulations require accurate understanding of the potential pollution cost. Furthermore, our work is helpful and instructive in environmental justice studies, especially in the local scale. It helps to identify the vulnerable populations in the complex urban environment.

Our results are counterintuitive, suggesting that air pollution positively influences housing prices in Oakland, CA. We believe that this counterintuitive result arises from two possible explanations. First, the results suggest that people may be insensitive to air quality if the overall ambient air quality is good, which is consistent with other literature we reviewed. Second, our study focused on a relatively small study domain, where the variability of air pollution concentrations and housing prices is low. The low variability of variables may lead to significant result even though the true influence is not significant.

Our results indicate that a larger, multi-regional study is probably the best way to determine the relationship between air pollution and housing prices. This study was conducted based on the 2016 data. Recently, data from high-resolution air pollution measurements have been expanding quickly. The Google Earth Outreach team has conducted the high-resolution air pollution measurement in Houston, London, Copenhagen, and Amsterdam. These data may prove useful to better understand how air pollution affects housing prices. The framework and method of this paper can be applied to multi-regions when the coverage of the high-resolution air pollution measurement is widely expanded. With more diversified regions covered in the analysis, we will be able to more accurately and precisely understand how air pollution concentrations influence housing prices.

## Figures and Tables

**Table 1:** Summary statistics of housing prices and air pollution concentration data.

	Housing Price, USD	NO Concentration, ppb	NO_2_ Concentration, ppb	BC Concentration, µg/m^3^
Sample size	26,386 ^a^	26,210 ^a^	26,210 ^a^	26,210 ^a^
Mean	275,664.1	10.293	12.121	0.457
Median	227,788.4	6.632	9.883	0.393
Standard deviation	200,586.2	8.68	5.07	0.23

^a^ Some apartments have multiple stories at the same locations, which makes the number of air pollution data less than the number of housing price data.

**Table 2:** Moran’s I test results for housing prices and three pollutants.

	Housing Price	NO Concentration	NO_2_ Concentration	BC Concentration
Moran’s I test statistic	0.27643	0.98498	0.9927	0.99127
Analytical method *p*-value	<0.001	<0.001	<0.001	<0.001
Monte-Carlo-based *p*-value	<0.001	<0.001	<0.001	<0.001

**Table 3:** Results of models with different pollutants ^a^.

Variables	NO Concentration	NO_2_ Concentration	BC Concentration
Intercept	2.9196 *** (0.4688)	2.5027 *** (0.45954)	2.8232 *** (0.46502)
Year Built	−0.0070745 *** (0.00033103)	−0.0068693 *** (0.00032984)	−0.0070433 *** (0.0003305)
Effective Year Built	0.010137 *** (0.0003401)	0.010268 *** (0.00033955)	0.010166 *** (0.00033986)
Construction type: concrete	−0.014669 (0.061875)	−0.0076211 (0.061662)	−0.0041038 (0.061783)
Construction type: frame	−0.3531 *** (0.020988)	−0.32364 *** (0.021187)	−0.34251 *** (0.021)
Construction type: masonry	−0.36805 *** (0.075532)	−0.32321 *** (0.074869)	−0.35448 *** (0.075232)
Other rooms: gym	−0.10416 ** (0.043856)	−0.080572 * (0.04356)	−0.092731 ** (0.043693)
Other rooms: office	0.17428 (0.40627)	0.18374 (0.4051)	0. 18428 (0.40593)
Parking type: Carport	−0.027576 (0.029369)	−0.016681 (0.029342)	−0.020942 (0.029382)
Parking type: garage	0.051695 *** (0.010082)	0.061715 *** (0.010229)	0.055929 *** (0.010152)
Parking type: Mixed	−0.0064772 (0.041319)	0.0046338 (0.041243)	−0.00011624 (0.04131)
Stories	0.020611 *** (0.0021083)	0.017629 *** (0.0021295)	0.020372 *** (0.0021063)
Rooms	−0.0095808 ** (0.0040749)	−0.0096307 ** (0.0060424)	−0.094721 ** (0.0040706)
Beds	−0.010024 (0.0064244)	−0.0092185 (0.0064047)	−0.010209 (0.0064193)
Baths	0.084969 *** (0.0086318)	0.082202 *** (0.0086111)	0.08463 *** (0.0086243)
Total area	0.00027102 *** (0.000014127)	0.00026972 *** (0.000014055)	0.0002711 *** (0.000014107)
Population density	0.000014708 *** $(2.7835\times 10^{-6}$)	0.00017308 *** $(2.8254\times 10^{-6}$)	0.000018455 *** $(2.9621\times 10^{-6}$)
Median income	$5.0184\times 10^{-6}$ *** $(3.0363\times 10^{-7}$)	$5.244\times 10^{-6}$ *** $(2.9925\times 10^{-7}$)	$5.3215\times 10^{-6}$ *** $(2.9968\times 10^{-7}$)
Non-employment rate	0.07601 (0.050197)	0.031651 (0.050804)	0.081003 (0.04948)
NO concentration	0.0054361 *** (0.00082701)	-	-
NO_2_ concentration	-	0.013246 *** (0.0016209)	-
BC concentration	-	-	0.22871 *** (0.03212)
lambda	0.21710 *** (0.019776)	0.18774 *** (0.020526)	0.20761 *** (0.020015)
R^2^	0.3183	0.3175	0.3178

^a^ *** significant at less than 0.1%, ** significant at less than 5%, * significant at 10%. (): standard error.

**Table 4:** Literature review summary.

Location	Air Pollution Concentrations									Method	Air Pollution Impact on Housing Price
	CO, µg/m^3^	NO_2_, µg/m^3^	O_3_, µg/m^3^	PM_2.5_, µg/m^3^	PM_10_, µg/m^3^	SO_2_, µg/m^3^	TSP, µg/m^3^	BC, µg/m^3^	NO, µg/m^3^		
Seoul, Korea (Kim & Yoon, 2019)					45.611					SDM	insignificant
Seoul, Korea (C.W. Kim, Phipps, & Anselin, 2003)		45.57 ^a^								SLM, SEM	insignificant
						82.95					negative
18 districts in Warsaw, Poland (Ligus & Peternek, 2017)		__			__					Linear, Logarithm, SLM, SEM	insignificant ^b^
Beijing, China (Mei, et al. 2020)	1399.1									Fixed effect	negative
		60.34									negative
			53.66								positive
				88.24							negative
					111.27						negative
						20.5					negative
286 prefectural cities in China (Chen & Jin, 2019)				64.81						IV	negative
288 Chinese cities (Huang & Lanz, 2018)					77.44					IV and discontinuity regression	negative
3 largest cities in Mexico (Gonzalez, Leipnik & Mazumder, 2013)					38.5, 51.7, 84					IV	negative
Metro areas US (Bayer et al., 2009)					42.21 (1990), 33.87 (2000)					IV	negative
All counties in USA (Chay & Greenstone, 2005)							64.1 (1970), 56.3 (1980)			quasi-experimental discontinuity regression	negative
Lebanon (Marrouch & Sayour, 2021)					27.67					Fixed effect	negative
Oakland, CA, USA		22.79						0.457	12.86	IV and SLM	positive

^a^ Paper reported NOx concentration in ppb and we converted it to µg/m^3^ with NO_2_ molecular weight. ^b^ Insignificant in most districts, some districts are positive or negative.

## Data Availability

Restrictions apply to the availability of housing price data. Housing price data were obtained from Estated, Inc. (https://estated.com/ accessed on 13 August 2020) and are available from the corresponding author with the permission of Estated, Inc. Air pollution data are publicly available in the supplementary material of the paper at https://pubs.acs.org/doi/10.1021/acs.est.7b00891 accessed on 13 August 2020. Social demographic data are from the 2016 American Community Survey, which is publicly available at https://www.census.gov/programs-surveys/acs accessed on 13 August 2020.
